# epiflows: an R package for risk assessment of travel-related spread of disease

**DOI:** 10.12688/f1000research.16032.3

**Published:** 2019-09-12

**Authors:** Paula Moraga, Ilaria Dorigatti, Zhian N. Kamvar, Pawel Piatkowski, Salla E. Toikkanen, VP Nagraj, Christl A. Donnelly, Thibaut Jombart

**Affiliations:** 1 Department of Mathematical Sciences, University of Bath, Bath, BA2 7AY, UK; 2 MRC Centre for Global Infectious Disease Analysis, Department of Infectious Disease Epidemiology, School of Public Health, Imperial College, London, W2 1PG, UK; 3 International Institute of Molecular and Cell Biology, Warsaw, Poland; 4 National Institute for Health and Welfare, Helsinki, Finland; 5 School of Medicine, Research Computing, University of Virginia, Virginia, USA; 6 Department of Statistics, University of Oxford, Oxford, OX1 3LB, UK; 7 Department of Infectious Disease Epidemiology, London School of Hygiene and Tropical Medicine, London, UK

**Keywords:** disease surveillance, outbreaks, epidemics, infectious, R, RECON

## Abstract

As international travel increases worldwide, new surveillance tools are needed to help identify locations where diseases are most likely to be spread and prevention measures need to be implemented. In this paper we present *epiflows*, an R package for risk assessment of travel-related spread of disease. *epiflows* produces estimates of the expected number of symptomatic and/or asymptomatic infections that could be introduced to other locations from the source of infection. Estimates (average and confidence intervals) of the number of infections introduced elsewhere are obtained by integrating data on the cumulative number of cases reported, population movement, length of stay and information on the distributions of the incubation and infectious periods of the disease. The package also provides tools for geocoding and visualization. We illustrate the use of *epiflows* by assessing the risk of travel-related spread of yellow fever cases in Southeast Brazil in December 2016 to May 2017.

## Introduction

Infectious disease outbreaks cause significant suffering and mortality in the affected populations, and damage the health, social and economic well-being of the families affected by diseases as well as producing significant economic costs for local and national governments. As we have seen with Ebola and SARS, disease outbreaks can spread beyond national borders ^1^. Travelers can acquire a disease while staying in a foreign country, and then seed new outbreaks in their home country after their return. As international travel increases worldwide, new surveillance tools are needed to help identify locations where diseases are most likely to be spread and prevention measures need to be implemented. This is essential to limit the global spread of local outbreaks.

Recently, Dorigatti *et al.* ^2^ developed a method to assess the risk of travel-related international spread of disease by integrating epidemiological and travel (by air, land and water) volume. The model developed by Dorigatti *et al.* ^2^ estimates the expected number of infections introduced elsewhere by taking into account population flows, lengths of stay, as well as the variability of the disease incubation and infectious periods. The method was applied to quantify the risk of spread of a recent outbreak of yellow fever in Southeast Brazil in December 2016 to May 2017, and was able to identify the countries that could have received travel-related disease cases capable of seeding local transmission.

In this paper we present epiflows, an R package that implements the method presented by Dorigatti *et al.* ^2^ for risk assessment of travel-related spread of disease. Using data on population movement between the location that is source of the infection and other locations, lengths of stay, as well as information about the disease incubation and infectious period distributions, the package allows the estimation of the number of (symptomatic and/or asymptomatic) infections that could be spread to other locations together with uncertainty measures. The package also provides tools for geocoding and visualization of population flows.

The remainder of the paper is organized as follows. First, we briefly describe the modelling framework that is implemented in the epiflows package. Second, we introduce the main components of epiflows including instructions for installation and main functions. Third, we illustrate the use of the package via the assessment of the risk of travel-related spread of yellow fever cases due to population flows between Southeast Brazil and other countries in December 2016 to May 2017. Specifically, we discuss the data required and show how to perform the statistical analyses, how to interpret the results, and the visualization options. Finally, the conclusions are presented.

## Model

In this Section we explain the modelling framework presented in 2 for estimating the expected number of infections departing from one infectious location during the incubation or infectious periods. These cases comprise exportations and importations. Exportations refer to the infected residents of the infectious location (i.e. location with sustained disease transmission) that travel to other locations. Importations (also referred to as returning travelers) are people that are infected during a temporary stay in the infectious location and then return to their home location. The following Sections describe how to model exportations and importations to produce the total number of expected cases that could be spread to other locations together with uncertainty measures.

### Exportations

Let *C __S,W__* denote the cumulative number of infections in location *S* in time window *W*. Here, *W* denotes the temporal window between the first and the last disease case in location *S*. Note that Dorigatti *et al.* ^2^ calculated *C __S,W__* by multiplying the number of confirmed and reported yellow fever cases by 10 to account for underreporting of asymptomatic and mild yellow fever cases.

Let *pop __S__* be the resident population of the infectious location *S*, and $T_{S,D}^{W}$ the number of residents of location *S* travelling to location *D* in time window *W*. The per capita probability that a resident from the infectious location travelled to other location *D* during the time window *W* is given by

$$p_{D}=\frac{T_{S,D}^{W}}{pop_{S}}\cdot$$

We assume that the incubation period ( *D __E__*) and the infectious period ( *D __I__*) are random variables, with associated probability distributions that are disease-specific. Using these, we can calculate the probability *p __i__* that an infection incubated or is infectious in time window *W* as

$$p_{i}=\mathrm{minimum}\left(\frac{D_{E}+D_{I}}{W},1\right)\cdot$$

Finally, the number of residents of the infectious location *S* that are infected and travel abroad during their incubation or infectious period during the time window *W* can be calculated as

*E __S,D__* = *C __S,W__* × *p __D__* × *p __i__.*

That is, *E __S,D__* is a product of the cumulative number of infections in location *S* in time window *W*, the per capita probability that a resident of *S* travels to location *D*, and the probability that an infection incubated or is infectious in time window *W*.

Note here that if travel data are expressed annually $\left(T_{S,D}^{A}\right)$ instead of in the time window *W*, travel data in the time window can be obtained as $T_{S,D}^{W}$ = ( $T_{S,D}^{A}$ × *W*)/365.

### Importations

Let $T_{O,S}^{W}$ be the number of travelers visiting location *S* from location *O* in time window *W*, and let *L __O__* denote the average length of stay. The per capita risk of infection of travelers visiting location *S* during their stay can be calculated as

$$\lambda_{S}=\frac{C_{S,W}\times L_{O}}{pop_{S}\times W}\cdot$$

The probability of returning to the home location while incubating or infectious is given by

$$p_{l}=\mathrm{minimum}\left(\frac{D_{E}+D_{I}}{L_{O}},1\right)\cdot$$

Finally, the expected number of travelers infected during their stay in the infectious location and returning to their home location before the end of the infectious period can be calculated as the product of the number of travelers, the per capita risk of infection and the probability of returning home while incubating or infectious,

$$I_{S,O}=T_{O,S}^{W}\times \lambda_{S}\times p_{l}\cdot$$

Note that, similarly to exportations, if travel data are expressed annually ( $T_{O,S}^{A}$) instead of in the time window *W*, travel data in the time window can be obtained as $T_{O,S}^{W}$ = ( $T_{O,S}^{A}$ × *W*)/365.

### Total number of exportations and importations

Finally, the expected number of infections departing from the infectious location *S* to location *O* during the incubation or infectious periods can be computed as the sum of the number of infected residents of *S* travelling during their incubation or infectious periods, and the travelers from abroad that are infected during their stay in *S* and return to their origin location before the end of the infectious period. That is,

$$T_{S,O}=E_{S,O}+I_{S,O\cdot }$$

Average estimates and the relative uncertainty are calculated by taking into account the variability of the incubation and infectious periods. Specifically, the method samples a large number of times from the incubation and infectious distributions, which produces a full distribution for *p __i__* (the probability that a disease case is incubated or infectious in the time window considered) and *p __l__* (the probability of returning to the home location while incubating or infectious). This, in turn, creates variability in exportations *E __S,O__* and importations *I __S,O__*, and finally in the total number of infections introduced in location O, *T __S,O__*.

## Methods

### Implementation

The R package epiflows [21] is hosted in the Comprehensive R Archive Network (CRAN) which is the main repository for R packages: http://CRAN.R-project.org/package=epiflows. Users can install epiflows in R by executing the following code:


install.packages("epiflows")


There is also a development version from GitHub which can be accessed at https://github.com/reconhub/epiflows. This version of the package may contain new features which are not incorporated in the version on CRAN yet but may be useful for some users. GitHub also includes issue tracking where users can note problems or suggestions for improvements. This development version from GitHub can be installed by using the install_github() function from the R package devtools ^3^:


install.packages("devtools")       
library("devtools")                
install_github("reconhub/epiflows")


When installing epiflows, other R packages which epiflows depends on are also automatically installed. These packages include sp ^4^ for manipulating spatial objects; geosphere ^5^ for calculating distances between locations; and leaflet ^6^ for visualization.

### Operation

The main function of the package is estimate_risk_spread() which calculates the mean and 95% confidence intervals of the number of cases spread to different locations from an infectious location. It is also possible to use this function to produce a data frame with all simulations (not just the mean and 95% confidence intervals that is computed from the simulations). This permits the user to aggregate the estimates and calculate confidence intervals with different levels using single simulations. To execute this function the following information is needed:

- population of the infectious location,
- number of infections in the infectious location, and the first and last dates of reported cases,
- number of travelers between the infectious location and other locations,
- average length of stay of travelers from other locations visiting the infectious location,
- distributions of the incubation and infectious periods,
- number of simulations to be drawn from the incubation and infectious period distributions,
- logical value indicating whether the returned object should be a data frame with all simulations, or a data frame with the mean and lower and upper limits of a 95% confidence interval of the number of infections spread to each location.

Other useful functions are plot() which produces visualizations of population flows between locations, and add_coordinates() which finds the coordinates of the locations.

## Use cases

In this Section we provide an example on how to use epiflows to calculate the number of yellow fever cases spreading from south-east Brazil to other countries due to human movement. We show how to define the arguments of the estimate_risk_spread() function, interpret the results, and make visualizations with the population flows.

### Data

We use the data YF_flows and YF_locations which are contained in the epiflows package as data(YF_flows) and data(YF_locations), respectively. These data contain the population size, the assumed number of yellow fever infections, dates of first and last case reporting, number of travelers and length of stay for the states of Espirito Santo, Minas Gerais, Rio de Janeiro, Sao Paulo, and for the whole region of Southeast Brazil (which comprises the four states of Espirito Santo, Minas Gerais, Rio de Janeiro and Sao Paulo) in the period December 2016 to May 2017 [19], [20].

Following Dorigatti *et al.* ^2^, the total number of yellow fever infections in each of the Brazilian states was calculated by multiplying the cumulative number of confirmed yellow fever cases reported in 7 by 10 to account for underreporting of asymptomatic and mild yellow fever cases. The dates of first and last case reported in each state were derived as described by Dorigatti *et al.* ^2^. Population data were obtained from the Brazilian Institute of Geography and Statistics website ^8^. These data also contain the number of travelers in the specified time window between the states of Espirito Santo, Minas Gerais, Rio de Janeiro, Sao Paulo (and the whole Southeast Brazilian region) and other countries. These estimates were obtained from World Tourism Organization data on the volume of air, land and water border crossings for Brazil for the year 2015 ^9^, having assumed that travelers were distributed across the Brazilian states according to the relative population density and having accounted for information on the monthly distribution of tourism and on the average duration of stay of international visitors to Brazil ^10^, as detailed in 2.

### The epiflows object

To aid in data organization between flows and metadata, we have implemented the epiflows object. This inherits the epicontacts object from the **epicontacts** package ^11^, storing three elements:

1. flows — a data frame defining the number of cases flowing from one location to another
2. locations — a data frame listing the locations present in flows and relevant metadata.
3. vars — a dictionary mapping column names in locations to known global variables defined in global_vars(). These global variables are used as default values in estimate_risk_spread().

Because a flow of cases from one location to another can be thought of as a contact with a wider scope, the epiflows object inherits from the epicontacts object, where locations are stored in the linelist element and flows are stored in the contacts element (though the user does not need to interact with these elements by name). By building on the epicontacts object, we ensure that all the methods for sub-setting an object of class epicontacts also applies to epiflows, reducing the maintenance effort.

An epiflows object can be created with the make_ epiflows() function by providing a data frame flows with the number of travelers between locations, a data frame locations with information about the locations, and the names of the columns of data frame locations indicating the name of each variable.

In the data frame flows each row represents the number of travelers from one location to the next. flows has at least three columns: columns from and to indicating where the flow starts and ends, respectively, and column n indicating the number of travelers that are in the flow. Data frame YF_flows contains the population flows of the Brazil data.


library("epiflows")



## epiflows is loaded with the following global variables in `global_vars()`:
## coordinates, pop_size, duration_stay, first_date, last_date, num_cases



data("YF_flows") 
head(YF_flows) 



##               from    to         n
## 1   Espirito Santo Italy  2827.572
## 2     Minas Gerais Italy 15714.103
## 3   Rio de Janeiro Italy  8163.938
## 4        Sao Paulo Italy 34038.681
## 5 Southeast Brazil Italy 76281.763
## 6   Espirito Santo Spain  3270.500


In data frame locations each row represents a location, and columns specify useful information about the locations such as ID, population, number of cases, dates and length of stay. locations must contain at least one column specifying the location ID used in the flows data frame. YF_locations contains, for each Brazilian state considered in our example, the code ( location_code), the population ( location_population), the number of assumed infections in the time window ( num_cases_time_window), and the dates of the first and last case reported ( first_date_cases and last_date_cases, respectively). It also contains length_of_stay which are the lengths of stay (in days) of the travelers visiting Brazil from other countries.


data("YF_locations")
head(YF_locations)



##      location_code location_population num_cases_time_window
## 1   Espirito Santo             3973697                  2600
## 2     Minas Gerais            20997560                  4870
## 3   Rio de Janeiro            16635996                   170
## 4        Sao Paulo            44749699                   200
## 5 Southeast Brazil            86356952                  7840
## 6        Argentina                  NA                    NA
##   first_date_cases last_date_cases length_of_stay
## 1       2017-01-04      2017-04-30             NA
## 2       2016-12-19      2017-04-20             NA
## 3       2017-02-19      2017-05-10             NA
## 4       2016-12-17      2017-04-20             NA
## 5       2016-12-17      2017-05-10             NA
## 6             <NA>            <NA>           10.9


Then, we can create an epiflows object called Brazil_epiflows as follows.


Brazil_epiflows <- make_epiflows(flows         = YF_flows,               
                                 locations     = YF_locations,           
                                 pop_size      = "location_population",  
                                 duration_stay = "length_of_stay",       
                                 num_cases     = "num_cases_time_window",
                                 first_date    = "first_date_cases",     
                                 last_date     = "last_date_cases"       
)                                                                        


### Arguments of the estimate_risk_spread() function

The arguments that need to be specified in estimate_risk_spread() to calculate the cases or infections introduced in other countries are as follows. The first argument is an epiflows object containing the number of travelers between locations, the population size, the number of cases, and the first and last dates of reporting in the infectious location, and the average length of stay in days of travelers from other locations visiting the infectious location.

The second argument of estimate_risk_spread() is location_code which is a character string denoting the infectious location code. We also need to specify the incubation and infectious period distributions. Specifically, we need to provide functions with a single argument n that generate n random incubation and infectious periods. To do this, we can use random generation functions of distributions that are implemented in R including Normal rnorm(), LogNormal rlnorm(), Gamma rgamma(), Weibull rweibull(), and Exponential rexp(). Details about the meaning and arguments of these functions can be obtained by typing ? and the function name (e.g., ?rnorm). We should consider the literature carefully before deciding on appropriate distributions. Examples of systematic reviews of incubation period distributions are 12 and 13. In this example, we use the specified distributions and parameterisation following 14 and 15. Thus, we assume that the incubation period *D __E__* is log-normally distributed with mean 4.6 days and variance 2.7 days, and that the infectious period *D __I__* is normally distributed with mean 4.5 days and variance 0.6 days. We can define functions incubation() and infectious() as


incubation <- function(n) {
  rlnorm(n, 1.46, 0.35)    
}                          

infectious <- function(n) {
  rnorm(n, 4.5, 1.5/1.96)  
}                          


Argument n_sim is the number of simulations to be drawn from the incubation and infectious period distributions. It is recommended to use at least 1,000 simulations. The last argument of estimate_risk_spread() is return_all_simulations. This is a logical value indicating whether the returned object should be a data frame with all simulations ( return_all_simulations= TRUE), or a data frame with the mean and lower and upper limits of a 95% confidence interval of the number of infections spread to each location ( return_all_simulations = FALSE).

### Execution of the estimate_risk_spread() function

Once we have constructed the objects needed to call estimate_risk_spread() we can execute the function and obtain the estimated mean number of cases spread to each country and the 95% confidence intervals. The code to calculate the cases spread from Espirito Santo is the following:


set.seed(2018-07-25)                                         
res <- estimate_risk_spread(Brazil_epiflows,                 
                            location_code = "Espirito Santo",
                            r_incubation = incubation,       
                            r_infectious = infectious,       
                            n_sim = 1e5                      
)                                                            


The results returned by estimate_risk_spread() are stored in the res object. This is a data frame with the columns mean_cases indicating the mean number of cases spread to each location, and lower_limit_95CI and upper_limit_95CI indicating the lower and upper limits of 95% confidence intervals. The result object is shown below.


res



##                          mean_cases lower_limit_95CI upper_limit_95CI
## Italy                     0.2233656        0.1520966        0.3078136
## Spain                     0.2255171        0.1537452        0.3126801
## Portugal                  0.2317019        0.1565528        0.3383112
## Germany                   0.1864162        0.1259548        0.2721890
## United Kingdom            0.1613418        0.1195261        0.2089475
## United States of America  0.9253419        0.6252207        1.3511047
## Argentina                 1.1283506        0.7623865        1.6475205
## Chile                     0.2648277        0.1789370        0.3866836
## Uruguay                   0.2408942        0.1627681        0.3517426
## Paraguay                  0.1619724        0.1213114        0.1926966


**Figure 1.**
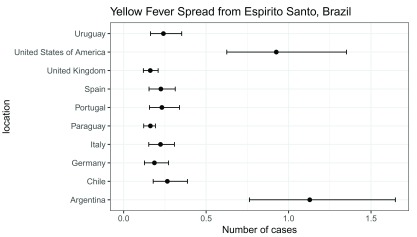
Mean number of yellow fever cases and 95% CI spread from Espirito Santo to other locations.

We can plot the results with ggplot() as follows ( Figure 1).


library("ggplot2")                                                                     
res$location <- rownames(res)                                                          
ggplot(res, aes(x = mean_cases, y = location)) +                                       
  geom_point(size = 2) +                                                               
  geom_errorbarh(aes(xmin = lower_limit_95CI, xmax = upper_limit_95CI), height = .25) +
  theme_bw(base_size = 12, base_family = "Helvetica") +                                
  ggtitle("Yellow Fever Spread from Espirito Santo, Brazil") +                         
  xlab("Number of cases") +                                                            
  xlim(c(0, NA))                                                                       


Note that if we set return_all_simulations equal to TRUE, the result object res will be a data frame with all simulations.


res <- estimate_risk_spread(Brazil_epiflows,                 
                            location_code = "Espirito Santo",
                            r_incubation = incubation,       
                            r_infectious = infectious,       
                            n_sim = 1e5,                     
                            return_all_simulations = TRUE    
)                                                            



head(res)



##          Italy     Spain  Portugal   Germany United Kingdom
## [1,] 0.1946102 0.1967196 0.2003120 0.1611614      0.1483634
## [2,] 0.2861083 0.2875748 0.3035947 0.2442577      0.1937063
## [3,] 0.1883587 0.1904003 0.1938773 0.1559844      0.1455385
## [4,] 0.2128377 0.2151446 0.2190734 0.1762560      0.1566001
## [5,] 0.2087285 0.2109909 0.2148439 0.1728531      0.1547432
## [6,] 0.2747205 0.2744030 0.2853804 0.2296033      0.1857099
##      United States of America Argentina     Chile   Uruguay  Paraguay
## [1,]                0.7999806 0.9754866 0.2289530 0.2082646 0.1552200
## [2,]                1.2124582 1.4784567 0.3470033 0.3156478 0.1837588
## [3,]                0.7742827 0.9441508 0.2215983 0.2015745 0.1502338
## [4,]                0.8749078 1.0668519 0.2503970 0.2277709 0.1619986
## [5,]                0.8580163 1.0462546 0.2455627 0.2233735 0.1609097
## [6,]                1.1397160 1.3897558 0.3261846 0.2967103 0.1790695


Using res, we can calculate the mean and 95% confidence intervals as follows.


meancases <- colMeans(res, na.rm = TRUE)                                   
quant     <- t(apply(res, 2, stats::quantile, c(.025, .975), na.rm = TRUE))
data.frame(mean_cases = meancases,                                         
           lower_limit_95CI = quant[, 1],                                  
           upper_limit_95CI = quant[, 2]                                   
)                                                                          



##			   mean_cases lower_limit_95CI upper_limit_95CI
## Italy		    0.2233975	     0.1522848	      0.3081296
## Spain		    0.2255621	     0.1539354	      0.3130456
## Portugal		    0.2317602	     0.1567465	      0.3388166
## Germany		    0.1864633	     0.1261107	      0.2725956
## United Kingdom	    0.1613646	     0.1196739	      0.2091694
## United States of America 0.9255753	     0.6259942	      1.3531231
## Argentina		    1.1286353	     0.7633297	      1.6499817
## Chile		    0.2648933	     0.1791584	      0.3872613
## Uruguay		    0.2409532	     0.1629695	      0.3522681
## Paraguay		    0.1619776	     0.1214615	      0.1928268


### Visualize population flows

We can visualize flows of people travelling between locations using plot() and passing as first parameter an epiflows object containing the population flows, and as second parameter the type of plot we wish to produce. Population flows can be displayed on an interactive map, as a network or as a grid between origins and destinations as described in the following sections.

### Flows displayed on an interactive map

We can visualize population flows on an interactive map using plot() with the parameter type="map". For this option to work, the epiflows object needs to include the longitude and latitude of the locations in decimal degree format. If coordinates are known, they can be added to the epiflows object using the add_coordinates() function from the epiflows package. In our example, the longitude and latitude data are in the data frame YF_coordinates.


data("YF_coordinates")
head(YF_coordinates)  

##		   id	    lon	      lat
## 1   Espirito Santo -40.30886 -19.18342
## 2	 Minas Gerais -44.55503 -18.51218
## 3   Rio de Janeiro -43.17290 -22.90685
## 4	    Sao Paulo -46.63331 -23.55052
## 5 Southeast Brazil -46.20915 -20.33318
## 6	    Argentina -63.61667 -38.41610


They can be added as follows.


Brazil_epiflows <- add_coordinates(Brazil_epiflows,                   
                                   coordinates = YF_coordinates[, -1])


If coordinates are unknown, we may resort to one of the freely available tools for geocoding. For example, we can use the geocode() function from the ggmap package ^16^. This function finds the latitude and longitude of a given location using either the Data Science Toolkit or Google Maps. We can also use add_coordinates() which uses geocode() to find the coordinates and directly add them to the epiflows object as follows.


Brazil_epiflows <- add_coordinates(Brazil_epiflows, overwrite = TRUE)


**Figure 2.**
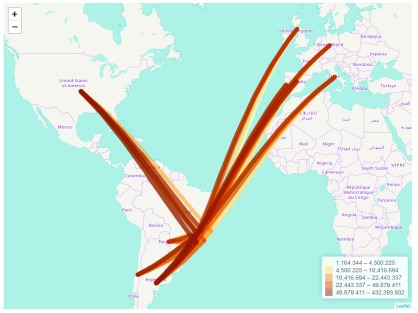
Population flows between Brazil states and other locations plotted using type = "map".

Once we have assigned coordinates to the epiflows object, we can use plot() with type="map" to visualize the population flows between locations in an interactive map ( Figure 2).


plot(Brazil_epiflows, type = "map")


The produced map can be zoomed and permits an easy examination of flows. plot() uses the gcIntermediate() function from the geosphere package ^5^ to obtain the great circle arcs between locations, and then uses the leaflet package ^6^ to create an interactive map with the connection lines. The connection lines are coloured according to flow volume, and as the mouse passes over the lines, lines highlight and information about connections is shown. We can also include parameters to specify a title, the center of the map or a color palette. An interactive version of this visualization is shown here: https://www.repidemicsconsortium.org/epiflows/articles/introduction.html#introduction-epiflows-map.

### Flows displayed as a network

**Figure 3.**
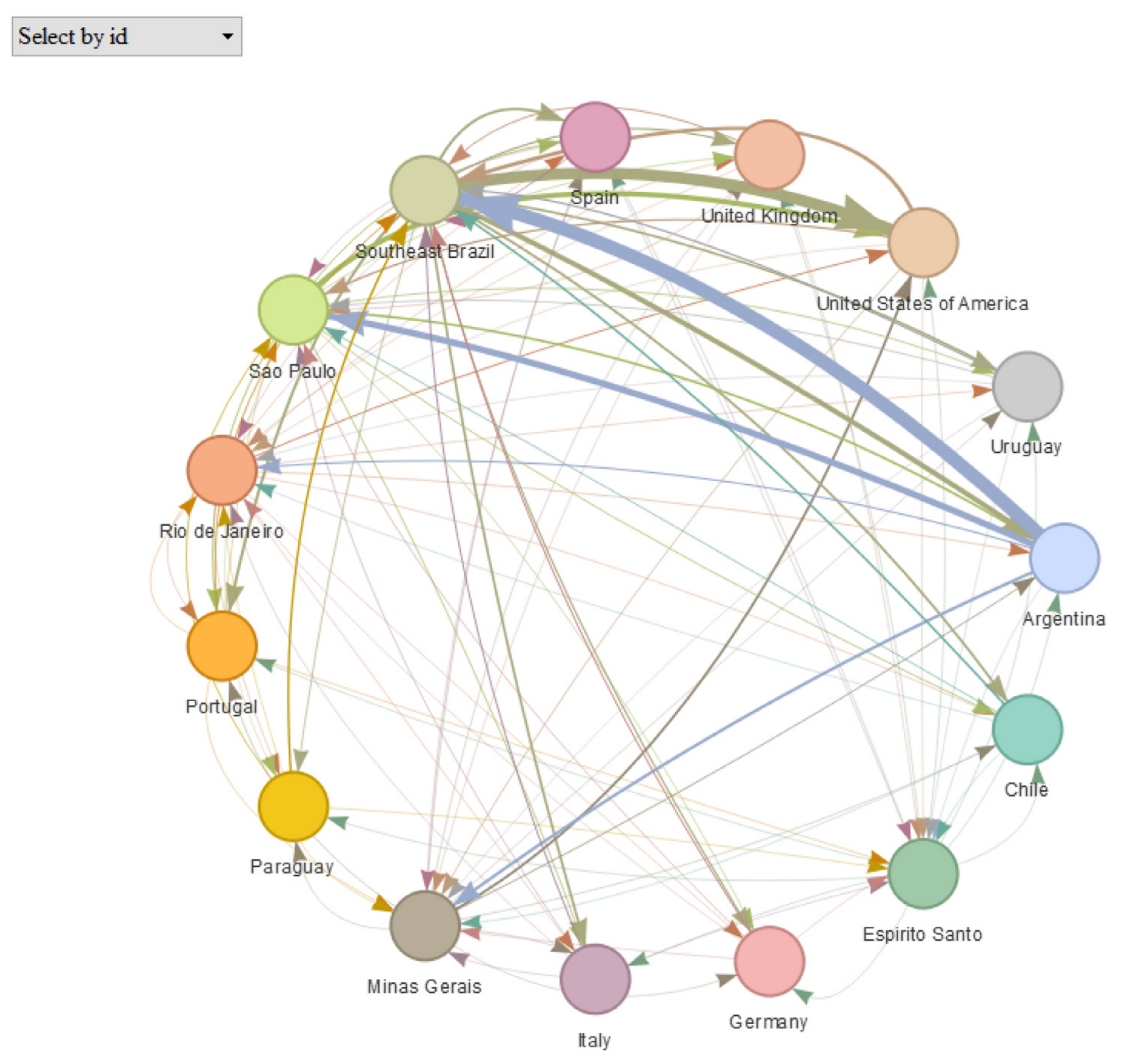
Population flows between Brazil states and other locations plotted using type = "network".

Population flows can also be displayed as a dynamic network using plot() with type = "network" ( Figure 3).


plot(Brazil_epiflows, type = "network")


This option uses the package visNetwork ^17^ to show the locations as nodes of a network and connections between them representing population flows. This plot is interactive and it is possible to highlight a given location and examine its population flows, as well as its population, number of cases, dates and length of stay. This type of plot can be used when coordinates of locations are missing. An interactive version of this plot can be viewed here: https://www.repidemicsconsortium.org/epiflows/articles/introduction.html#introduction-epiflows-vis.

### Flows displayed as a grid between origins and destinations

**Figure 4.**
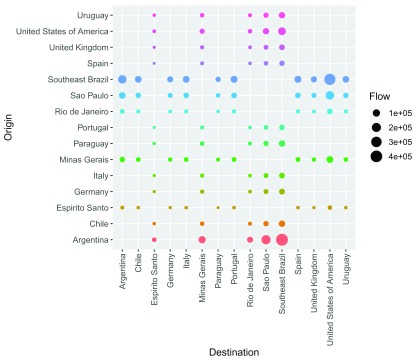
Population flows between Brazil states and other locations plotted using type = "grid".

Finally, population flows can also be shown as a grid between locations with the option type="grid" ( Figure 4).


plot(Brazil_epiflows, type = "grid")


This plot shows flows between locations as points by positioning origins and destination in y and x axes, respectively. When using this option, additional arguments can be passed to set the size, color or shape of the points as in function geom_point() of package ggplot2 ^18^. As the network plot, the grid plot can be used when coordinates of locations are missing.

Arrivals of non-resident tourists at Brazilian national borders by country of residence Annual volumes of air, land and water border crossings for Brazil relative to inbound tourism from years 2011 to 2015 obtained from the World Tourism Organisation (UNWTO). Click here for additional data file. Copyright: © 2019 Moraga P et al. 2019 Data associated with the article are available under the terms of the Creative Commons Zero "No rights reserved" data waiver (CC0 1.0 Public domain dedication).

Trips abroad by Brazilian resident visitors to countries of destination Annual volumes of air, land and water border crossings for Brazil relative to outbound tourism from years 2011 to 2015 obtained from the World Tourism Organisation (UNWTO). Click here for additional data file. Copyright: © 2019 Moraga P et al. 2019 Data associated with the article are available under the terms of the Creative Commons Zero "No rights reserved" data waiver (CC0 1.0 Public domain dedication).

## Summary

In this article we have presented the epiflows package for risk assessment of travel-related spread of disease. This package allows the estimation of the expected number of infections that could be introduced to other locations from the source of infection by integrating data on the number of cases reported, population movement, length of stay and information on the distributions of the incubation and infectious periods of the disease. The package also provides tools for geocoding and visualization which facilitate the interpretation of the results.

First, we presented how to estimate exportations, importations and total number of infections using the modelling framework introduced by Dorigatti *et al.* ^2^. Then, we demonstrated the use of the package by assessing the risk of travel-related spread of yellow fever cases in Southeast Brazil in December 2016 to May 2017. Specifically, we have shown how to construct an epiflows object containing population flows and information about locations, and how to use the function estimate_risk_spread() to obtain the average and confidence intervals of the estimated number of infections introduced elsewhere. Finally, we have shown how to visualize the results and produce maps of the population flows.

International travel has an important role in the spread of infectious diseases across national borders. We think the epiflows package represents a useful tool for disease surveillance that can help public health officials identify locations where diseases are most likely to spread and prevention measures are most needed.

## Data availability

The data referenced by this article are under copyright with the following copyright statement: Copyright: © 2019 Moraga P et al.

Data associated with the article are available under the terms of the Creative Commons Zero "No rights reserved" data waiver (CC0 1.0 Public domain dedication). 

**Dataset 1. Arrivals of non-resident tourists at Brazilian national borders by country of residence.** Annual volumes of air, land and water border crossings for Brazil relative to inbound tourism from years 2011 to 2015 obtained from the World Tourism Organisation. https://doi.org/10.5256/f1000research.16032.d215763 ^19^.

**Dataset 2. Trips abroad by Brazilian resident visitors to countries of destination.** Annual volumes of air, land and water border crossings for Brazil relative to outbound tourism from years 2011 to 2015 obtained from the World Tourism Organisation. https://doi.org/10.5256/f1000research.16032.d215765 ^20^.

## Software availability

1. Dedicated website for epiflows, including installation guidelines and documentation: https://www.repidemicsconsortium.org/epiflows
2. Software available from: https://cran.r-project.org/package=epiflows
3. Source code available from: https://github.com/reconhub/epiflows
4. Archived source code at time of publication: http://doi.org/10.5281/zenodo.1401806 ^21^.
5. Software license: MIT License
