# Supplementary material for: epiflows: an R package for risk assessment of travel-related spread of disease
Source: F1000Res. 2019 Sep 12;7:1374. Originally published 2018 Aug 31. [Version 3] doi: 10.12688/f1000research.16032.3 (PMC6738191; doi:10.12688/f1000research.16032.3)
Supplement: Peer review history

# Peer review: Reviewer response for version 2

**DOI:** 10.5256/f1000research.19559.r52017

**Published:** 2019-09-09

**Authors:** Noam Ross

**Affiliations:** 1 EcoHealth Alliance, New York City, NY, USA

The authors have addressed all concerns I raised in the first version of this paper. 

 I note one small error: `num_sim` is used in the text, but in the package and example code this argument is `n_sim`.

I confirm that I have read this submission and believe that I have an appropriate level of expertise to confirm that it is of an acceptable scientific standard.

# Peer review: Reviewer response for version 2

**DOI:** 10.5256/f1000research.19559.r52018

**Published:** 2019-09-05

**Authors:** Jon Zelner

**Affiliations:** 1 Department of Epidemiology, University of Michigan, Ann Arbor, MI, USA

These all appear to be useful/helpful changes; thanks to the authors!

I confirm that I have read this submission and believe that I have an appropriate level of expertise to confirm that it is of an acceptable scientific standard.

# Peer review: Reviewer response for version 1

**DOI:** 10.5256/f1000research.17509.r38978

**Published:** 2018-10-18

**Authors:** Jon Zelner

**Affiliations:** 1 Department of Epidemiology, University of Michigan, Ann Arbor, MI, USA

In this paper, the authors have presented and provided use-case examples for an R package that allows the estimation of the number of infections spread via travel, given information on the prevalence of disease in the sending location and the rate of flow between two locations. 

 Although the paper builds on a published method, it should provide some additional detail about the method in the introductory sections. Specifically, in the section titled "Exportations" on page 3, the authors say that "the method assumes that the incubation period T_E and the infectious period T_I follow specific probability distributions."

 More explanation of the modeling assumptions will allow potential users of this package to make an informed decision about whether it will be useful to them without going back to the original manuscript in which the method was described. In addition, the reuse of "T" to indicate the rate of travel in the paragraph above and the incubation and infectious periods is confusing and should be changed.

 From reading the paper and looking at the github repository, it seems like it is completely up to the user to specify the distribution of the incubation and infectious periods. One potentially helpful addition to the package would be the addition of data with estimates of these quantities for different pathogens where available, along with citations/dois for the data source. This would make this more user-friendly and amenable to rapid response.

I confirm that I have read this submission and believe that I have an appropriate level of expertise to confirm that it is of an acceptable scientific standard, however I have significant reservations, as outlined above.

# Response

**Published:** 2019-07-24

**Authors:** Paula Moraga

**Affiliations:** Lancaster University, UK

Thank you for your helpful and insightful comments. Please find our responses below.

 1. Thank you for pointing out the choice of incubation and infectious period distributions needs clarification. In Section "Exportations" we have mentioned we assume that the incubation period and the infectious period are random variables, with associated probability distributions that are disease-specific. We have also cited the papers we used to choice the incubation and infectious period distributions of our yellow fever example.

 2. Thank you for the notation review. Now we have changed the notation of the incubation period T_E by D_E, and infectious period T_I by D_I, where letter D stands for duration. Now letter T is only used for travelers.

 3. We agree on the importance of the choice distributions of incubation and infectious periods. In Section "Arguments of the estimate_risk_spread() function", we have added that we can define these distributions using random generation functions of distributions that are implemented in R. However, we decided not to provide data for incubation and infectious periods of other pathogens since we expect users to have some knowledge of the life history of the disease they want to apply it to. We have mentioned that users should consider the literature carefully before deciding on appropriate distributions, and we reference two review papers that may be useful.

# Peer review: Reviewer response for version 1

**DOI:** 10.5256/f1000research.17509.r38778

**Published:** 2018-10-09

**Authors:** Noam Ross

**Affiliations:** 1 EcoHealth Alliance, New York City, NY, USA

The authors present and provide a tutorial to epiflows, an R package for calculating the risk of travel-related disease export from an epidemic area.  It is a useful implementation of an algorithm, with associated visualization tools.  It is technically sound though the scaffolding around the core algorithm is somewhat over-engineered.  

 Both the package design and paper description imply this package is designed for rapid risk assessment.  My comments are primarily in regard to the clarity of the description and the usability of the package API in this context.

- The `global_vars()` function is a thin wrapper around R's `options()` mechanism that obfuscates what is actually happening,  and the name itself is somewhat confusing. (There are many different uses and abuses of global variables in R). It is difficult to see how this function improves over simply telling the user that default variables are defined by `epiflows.vars` in R's options mechanism (epiflows.varnames might be clearer).
- While it is mentioned that the `epiflows` object inherits from `epicontacts`, it is not clear what this means and how it is relevant to the user.  Given the epiflows object has different contents than the `epicontacts` object, it should be explained.  I see in the package vignette that subsetting methods are inherited, but given that the object contents are different, this should be demonstrated in the paper.  Otherwise it is hard to see what the advantage of this object is at all, over simply passing data frames to the algorithm function.
- The paper (as well as the code demonstration and vignette in the package), spends considerable time on the conversion of the partially processed data in `YF_Brazil` to data appropriately structured to be used in the `make_epiflows()` and `estimate_risk_spread()` function. This is confusing and not very useful - most users will not have data in exactly the format of `YF_Brazil`, and it distracts from the description of the core package functionality.  It would be far clearer to introduce and demonstrate the functions first using data in its ready-to-analyze form. If the authors wish to provide an example of an actual full workflow, they should start with actual raw source data from the the supplementary data. A useful addition would also be to describe possible sources of the data needed.
- The distributions of incubation and infectious periods is important and glossed over rather quickly here.  First, on page 3, the text reads, "The method assumes that the incubation period TE and the infectious period TI follow specific probability distributions."  This is unclear, I think "disease-specific" would be clearer.  Moreover, in the code tutorial, these distributions are simply assumed.  It would make more sense to describe why such distributions are selected and the source of the parameters, e.g., "For yellow fever we choose these distributions based on clinical literature describing the disease course as X to Y days of incubation and V to Z days infectious period (Citation 1, Citation 2).  Lognormal or Gamma distributions are typically used for these distributions..."
- It also confusing that both individuals traveling and incubation times are shown as T in the mathematical notation.

I confirm that I have read this submission and believe that I have an appropriate level of expertise to confirm that it is of an acceptable scientific standard, however I have significant reservations, as outlined above.

# Response

**Published:** 2019-07-24

**Authors:** Paula Moraga

**Affiliations:** Lancaster University, UK

Thank you for your thorough review and insightful comments. We address each comment separately below.

 1. In response to the comment regarding the `global_vars()`  function, we think this is a fair point. The main advantage of the use of `global_vars()`  is giving the user a way to easily recover the `epiflows.vars` option variables in the case that they made an error in specifying new variables (global_vars(reset = TRUE)).

 2. Thank you for bringing to our attention that the use of epicontacts needs an explanation. Because a flow of cases from one location to another can be thought of as a contact with a wider scope, the `epiflows` object inherits from the `epicontacts` object, where locations are stored in the "linelist" element and flows are stored in the "contacts" element (though the user does not need to interact with these elements by name).  By building on the epicontacts object, we ensure that all the methods for sub-setting an object of class `epicontacts` also applies to `epiflows`, reducing the maintenance effort. We have clarified this in Section “The epiflows object” of the manuscript.

 3. In order to present an example that is as clear as possible for readers, we have modified the yellow fever example and now we do not describe datasets that do not have ready-to-analyze form. Now we start by describing the YF_flows and YF_locations objects which can be directly passed to the make_epiflows() function to create the Brazil_epiflows object.

 4. Thank you for pointing out the importance of the distributions of incubation and infectious periods. In Section "Exportations" we have replaced "the method assumes that the incubation period T_E and the infectious period T_I follow specific probability distributions." by "We assume that the incubation period (D_E) and the infectious period (D_I) are random variables, with associated probability distributions that are disease-specific."  In Section "Arguments of the estimate_risk_spread() function", we have cited the papers we used to choose the incubation and infectious period distributions of the yellow fever example. We decided not to provide data for incubation and infectious periods of other pathogens since we expect users to have some knowledge of the life history of the disease they want to apply it to. We have also mentioned that users should consider the literature carefully before deciding on appropriate distributions, and we reference two review papers that may be useful.

 5. Thank you for the notation review. Now we have changed the notation of the incubation period T_E by D_E, and infectious period T_I by D_I, where letter D stands for duration. Now letter T is only used for travelers.
